# Looking for the best of both worlds

**DOI:** 10.7554/eLife.36366

**Published:** 2018-04-04

**Authors:** Bridget M Kuehn

**Affiliations:** eLifeChicagoUnited States

**Keywords:** parents, women in science, diversity, careers in science, family friendly policies

## Abstract

Research institutions could do more to support scientists who are pregnant or have young children.

“There is this deeply ingrained idea that it's impossible to do science and anything else,” says Ottoline Leyser, director of the Sainsbury Laboratory at the University of Cambridge. “Clearly, it's completely possible to have a scientific career and a family because men have been doing it for years.”

But how does one go about being a good scientist and a good parent? Last year Leyser worked with the Royal Society on a project called Parent Carer Scientist that highlights some 150 scientists in the UK who have combined a career in science with a family life. The importance of finding a supportive working environment was a central theme, along with advice to “learn to work smarter” and “don't be afraid to ask for help.”

In a foreword to the report Leyser also tried to dispel some of the myths about what it takes to be successful in science: “success becomes so narrowly defined that it looks unattractive to the most interesting and imaginative people, who are the very people science needs.” An optimal scientific community, she continues, will include people “weaving their research activities into their wider lives in different ways.”

The scientists highlighted in Parent Carer Scientist have all established themselves as researchers, but what is it like for people starting out on their careers?

## Parental leave and childcare

One of the biggest challenges facing a scientist who wants to start a family is that parenthood often coincides with being a graduate student or a postdoc, largely because most researchers do not secure their first permanent or independent position until they are in their thirties. “People can’t wait that long to have kids,” says Tracy Costello, who chairs the National Postdoctoral Association (NPA)’s board of directors.

John Carroll, dean of biomedical and psychological sciences at Monash University in Australia, is familiar with this issue: about 80% of the scientists who take maternity leave at Monash are postdocs. This creates a structural problem for science, he says, because the time and/or funding available for their training might be limited. It is also a time when many women leave the field. Taking maternity leave or a break during this training period can also result in women having fewer publications than their male counterparts. “It is critical,” he says, “that we try to minimize the impact of career breaks on researchers.”

A big problem in the US is a lack of paid maternity and paternity leave for postdocs. In 2014 the Center for WorkLife Law in San Francisco in partnership with the NPA collected data on postdocs from 66 institutions in the US: over half (53%) did not provide paid maternity leave to postdoc employees, and the numbers were worse for postdoc trainees (61%), individually funded postdocs (62%), and externally funded postdoc mothers (74%). And even when parental leave is available, it may be too short for women to fully recover. Many fathers also reported pressure to not take the paternity leave they were entitled to. And when parent scientists do return to work in the US, they find that childcare costs can eat up between 50 and 100% of their salaries.

Institutions that don’t have family friendly policies are driving potentially great scientists to other careers

The Center’s report, Parents in the Pipeline, also highlights another problem: the “hostility” of principal investigators to new mothers. The lack of maternity and paternity leave for postdocs in the US is also part of a wider debate started by the NPA and others about supporting the needs of postdocs more generally.

Costello says that a failure to support young scientists as they navigate training and parenthood also contributes to the attrition of minorities and individuals who are the first in their families to pursue academic careers: “Institutions that don’t have family friendly policies are driving potentially great scientists to other careers.”

Some countries offer paid parental leave and allow it to be shared between the mother and father. In the UK, for example, parents can share 52 weeks of paid leave after the birth or adoption of a child, and German parents can share 14 months of paid leave plus additional time if one or both parents work part-time. And government subsidized childcare is available in some parts of the world. In France, for example, subsidized infant care is available through crèches and children between the ages of three and six can attend preschool for free.

Some scientists may find that their care-giving responsibilities require them to pause their careers. In response to this a number of organizations – including CERN, the European particle physics laboratory in Geneva, and the Daphne Jackson Trust in the UK – have created full-time and part-time fellowships to help scientists re-enter the workforce after career breaks.

## Inexpensive options

Unlike paid parental leave or subsidized childcare, many parent-friendly benefits cost little or nothing, such as providing lactation rooms or scheduling lab meetings at times that don’t clash with taking children to and from school. “Most universities probably aren’t paying attention to all of the low or no-cost opportunities,” says Costello.

A casual lunchtime conversation with a fellow in the Africa Science Leadership Program led Smeetha Singh, a project coordinator for the program, to arrange for a larger room with a crib so that the fellow’s mother could come with her to a workshop to help care for her newborn. “I didn’t even think this was a big deal,” Singh recalls, “but she said ‘Thank you so much – this is a life-changing workshop, but I still get to bond with my baby, to breastfeed my baby’.”

Flexible work schedules are another no-cost way that institutions can support families. For example, says Costello, institutions can allow scientists to work from home when they are writing, reading or doing other tasks that do not require them to be at the bench. Such flexibility also allows many couples to co-ordinate their working hours so that, for example, one goes into work early and the other goes in late. “The flexibility that science brings is much greater than lots of jobs,” says Leyser.

In addition to no-cost options, such as providing rooms, Singh’s organization is also making it easier for parents in the leadership program to travel by paying for meals for caregivers who travel with the fellow and their child. Other organizations offer similar benefits: the Society for Molecular Biology & Evolution, for example, offers subsidized childcare for its annual meeting and travel awards to help defray meeting-related childcare costs. Singh recommends that when societies and other institutions organize meetings, they should talk to the venue about amenities like larger rooms or childcare.

Given the importance of conferences in the research community, Rebecca Calisi of the University of California, Davis and a Working Group of Mothers in Science recently published an article, ‘How to tackle the childcare-conference conundrum’, that listed a number of actions that conference organizers can take to address some of the challenges that conferences present for pregnant women and parents. “We have compiled four concrete suggestions we call CARE (for Childcare, Accommodate families, Resources, Establish social networks),” wrote Calisi and her colleagues. The suggestions range from parent/caregiver social networks to onsite childcare facilities, such as those provided by some very large conferences like the annual meeting of the Society for Neuroscience.

## Improving the workplace environment

What can be done to make research institutions more supportive to scientists who are pregnant or have young children? “Everybody’s needs are different,” says Leyser, “so I think the most important thing is to try and build an environment where people feel free to say what their needs are.”

Too often women fear a backlash if they ask for even minor accommodations, such as avoiding harmful chemicals during pregnancy. The Parents in the Pipeline report found that only 40% of postdoctoral mothers requested pregnancy accommodations. Instead, one woman reported she delayed telling her colleagues about her pregnancy until she couldn’t hide it any more. Others reported that their new parent status resulted in hostility from their principal investigators, and people of color were nearly twice as likely to report this type of hostility as their white peers (25% vs 14%). However, when mothers were willing to ask for accommodations, their requests were fulfilled 93% of the time. “Those who are willing to stand up and ask for it, got what they wanted,” says Costello.

Some institutions have turned to organized approaches to promoting workplace environments that are welcoming and supportive. In Australia, for example, Monash University is one of 40 institutions that are pursuing recognition through the Athena SWAN Charter – a scheme that was established in the UK in 2005 to promote gender equality in science – under the auspices of the program. As a first step, the institutions have to assess their existing environment. Already, Carroll and his colleagues are working to make returning from maternity leave “as seamless as possible”, which will involve continuing to offer onsite childcare and having lactation rooms easily accessible.

Singh and her colleagues ensure that 50% of the participants in Africa Science Leadership Program are women, and encourage those who are pregnant or new parents to apply: “we want to be very clear that you are welcome if you have a new baby, please do apply.”

## Note

This Feature Article is part of the Scientist and Parent collection.

